# *Gluconobacter Oxydans*-Based MFC with PEDOT:PSS/Graphene/Nafion Bioanode for Wastewater Treatment

**DOI:** 10.3390/bios12090699

**Published:** 2022-08-31

**Authors:** Sergei Tarasov, Yulia Plekhanova, Vadim Kashin, Pavel Gotovtsev, Maria Assunta Signore, Luca Francioso, Vladimir Kolesov, Anatoly Reshetilov

**Affiliations:** 1 G.K. Skryabin Institute of Biochemistry and Physiology of Microorganisms, Pushchino Center for Biological Research of the Russian Academy of Sciences, Moscow Region, 142290 Pushchino, Russia; 2 FSBIS V.A. Kotelnikov Institute of Radio Engineering and Electronics, Russian Academy of Sciences, 125009 Moscow, Russia; 3 Biotechnology and Bioenergy Department, National Research Centre “Kurchatov Institute”, 123182 Moscow, Russia; 4 Moscow Institute of Physics and Technology (National Research University), Moscow Region, 141701 Dolgoprudny, Russia; 5 CNR IMM, Institute for Microelectronics and Microsystems, Via Monteroni, I-73100 Lecce, Italy

**Keywords:** microbial fuel cell, *Gluconobacter oxydans*, PEDOT:PSS, graphene, Nafion, boost converter accumulation, wastewater treatment

## Abstract

Microbial fuel cells (MFCs) are a variety of bioelectrocatalytic devices that utilize the metabolism of microorganisms to generate electric energy from organic matter. This study investigates the possibility of using a novel PEDOT:PSS/graphene/Nafion composite in combination with acetic acid bacteria *Gluconobacter oxydans* to create a pure culture MFC capable of effective municipal wastewater treatment. The developed MFC was shown to maintain its activity for at least three weeks. The level of COD in municipal wastewater treatment was reduced by 32%; the generated power was up to 81 mW/m^2^ with a Coulomb efficiency of 40%. Combining the MFC with a DC/DC boost converter increased the voltage generated by two series-connected MFCs from 0.55 mV to 3.2 V. A maximum efficiency was achieved on day 8 of MFC operation and was maintained for a week; capacitors of 6800 µF capacity were fully charged in ~7 min. Thus, *G. oxydans* cells can become an important part of microbial consortia in MFCs used for treatment of wastewaters with reduced pH.

## 1. Introduction

Wastewater treatment will be one of the most important tasks for humanity in the near future [1,2,3]. The use of traditional technologies (e.g., coagulation, adsorption and ion exchange membranes) for wastewater treatment does not always contribute to the complete removal of pollutants, so they re-enter the environment [4]. The need still exists for methods suitable for long-term effective and sustainable use. One of such methods is microbial fuel cell (MFC) technology. MFCs are energy conversion devices containing electrogenic bacteria capable of harvesting electrical energy directly from organic waste while simultaneously removing pollutants [5]. Oxidation of organic substrates by microbes takes place in the anode compartment and leads to the formation of carbon dioxide, protons and electrons [6]. Electrons formed during the oxidation of substrates enter the cathode via an external circuit and are combined there with protons (entering through a proton-permeable membrane from the anode compartment) and oxygen; this leads to the generation of an electric current [7].

MFCs attract the attention of researchers because of their unique properties useful in wastewater treatment. They do not require external energy sources, can operate at low concentrations of pollutants and can be used under unfavorable external conditions in treatment of various wastewater compositions [8]. Separate microorganisms can be used, such as *Shewanella* sp. [9], *Geobacter* sp. [10], various yeasts [11,12] and microbial consortia, that help to clean wastewater from complex-composition pollutants [13,14,15]. Operation of the MFC requires the presence of at least one electrogenic microorganism in it. Hundreds of electricigens have been isolated and used in MFCs to date, but numerous microorganisms capable of generating electricity are still waiting to be discovered [16]. Nevertheless, investigators often use not individual microorganisms but microbial consortia because a variety of electricigens in most cases contributes to more efficient current generation [17]. The best performing MFCs always use mixed communities—such as wastewater communities or activated sludge—as anode biocatalyst [18,19]. Moreover, pure cultures of electricigens require relatively strict working conditions and can process only certain substrates, whereas mixed consortia are more suitable for the use of complex substrates [20]. Nevertheless, pure cultures are very useful for choosing electrode materials, redox mediators and conditions improving electron transfer in the system, as well as for studying the possibilities of reducing the number of microbial strains in mixed cultures with operational efficiency preserved [21]. Moreover, a pure culture MFC can be considered as a means of studying the metabolism of bacteria themselves, the mechanism of electron transfer, patterns of direct and mediator transfer in the process of bioelectrocatalysis [22,23].

Only some microorganisms are able to transfer electrons directly to the electrodes via cytochromes located in the outer membrane, or via conductive pili and nanowires naturally occurring on the surface of such bacteria [24]. Electron transfer between microbial cells and electrodes can also be carried out via redox compounds produced by the cells themselves [25] or added into the MFC compartment manually [26]. Gram-negative bacteria of the genus *Gluconobacter* are frequently used for the development of electrodes for biosensors and microbial fuel cells [27] because they have a wide substrate specificity and are capable of transferring electrons fairly quickly. In particular, *G. oxydans* cells are a promising candidate for the creation of effective MFCs because their cell membrane contains pyrroloquinolinquinone (PQQ)-dependent dehydrogenases, which are capable of efficiently oxidizing a number of compounds [28]. Our previous work has developed a new conductive composite based on PEDOT:PSS, graphene and Nafion, which we used to effectively immobilize *G. oxydans* bacteria on the surface of screen-printed graphite electrodes [29]. It has been shown that the use of PEDOT:PSS in combination with graphene improves the analytical characteristics of a microbial biosensor; the use of graphene neutralizes the negative action of PEDOT:PSS on bacterial cells. Nafion as an immobilizing agent makes it possible to achieve an impressive bioelectrode stability of more than 120 days. The use of this conductive composite in combination with *G. oxydans* cells in an MFC can be appropriate for creating a device capable of long-term low-pH wastewater treatment and simultaneous efficient electric energy generation. Moreover, these microorganisms are used in food [30,31], pharmaceutical [32,33] and cosmetic [34] industries, so they can be also used in wastewater treatment systems of these biotechnological enterprises.

The aim of this work was to create an MFC based on a *G. oxydans* pure culture immobilized on the surface of graphite bioanodes by a PEDOT:PSS/graphene/Nafion conductive composite. Experiments were carried out to analyse the spectral and electrochemical characteristics of the formed electrodes, their long-term stability and ability to generate electricity during the treatment of municipal wastewater samples. A DC/DC boost converter was used for harvesting the produced electric energy to enable increasing the generated voltage from ~0.4 V to 3.2 V, which expanded the applicability of these devices in wastewater treatment systems.

## 2. Materials and Methods

### 2.1. Reagents

Monopotassium phosphate, sodium hydroxide, sodium chloride, anhydrous acetic acid, D-glucose (Mosreaktiv, Moscow, Russia); 2,6-dichlorophenolindophenol sodium salt dihydrate, Nafion 117 (5% solution), graphene/PEDOT:PSS hybrid ink, bacteriological agar, potassium ferricyanide (Sigma, Burlington, MA, USA); ethanol, glycerol, sorbitol, yeast extract and potassium chloride (Dia-M, Moscow, Russia) were used. Graphite electrodes (S-3M, OOO Poliprof-L, Moscow, Russia) served as working electrodes

### 2.2. Anode Fabrication

The strain *Gluconobacter oxydans* sbsp. *industrius* VKM B-1280 (All-Russian Collection of Microorganisms) was used as a biocatalyst for MFC. *G. oxydans* are gram-negative aerobic bacteria and were cultivated according to the methodology outlined in [35]. After the cultivation, the cell suspension was rinsed and diluted to a concentration of 1 mg wet weight per μL with a phosphate buffer (PBS).

Graphite rods (65 mm in height and 6 mm in diameter) were used as an anode and a cathode for MFCs.

Graphene/PEDOT:PSS hybrid ink was applied to the graphite rod and dried for 12 h at room temperature. The concentration of PEDOT:PSS was 13 ng/mm^2^ and that of graphene, 64.8 ng/mm^2^. Then, cells were mixed at a ratio of 5:1 (*v*/*v*) with a Nafion 117 solution. The produced mixture was sonicated for a total of 1 min. After that, the mixture was deposited on the working electrode surface and allowed to dry at an ambient temperature for 1 h. Then the electrode was left at +4 °C for 12 h. The concentration of bacterial cells on the electrode surface was 40 μg/mm^2^. Prior to the first measurement, the fabricated electrode was kept in a PBS for 30 min to decrease the initial drift time and to stabilize the electrode signal.

### 2.3. MFC Setup and Operation

**Figure 1:**
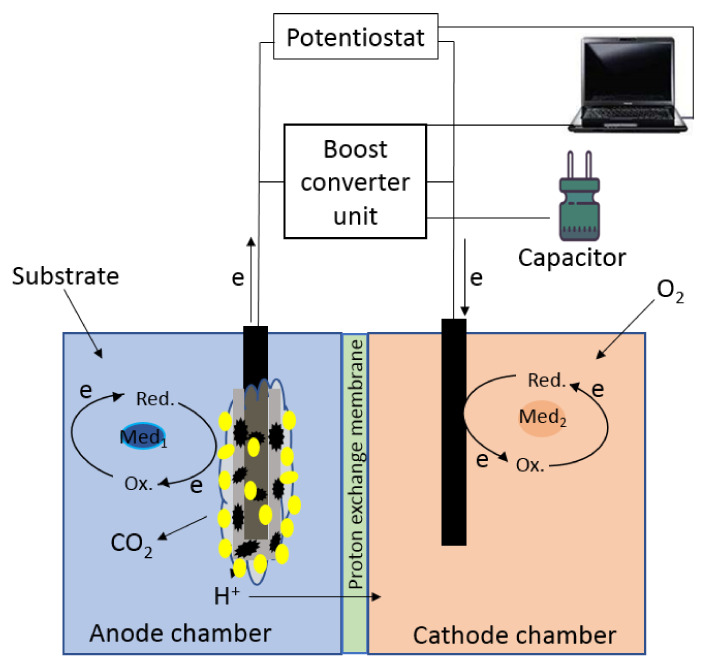
A schematic diagram of MFC operation and external connections.

A dual chambered MFC was made from an acrylic block (65 × 46 × 70 mm^3^) using two interconnected cuvettes, each with a working volume of 50 mL. The chambers were separated by an MF-4SK proton-selective membrane (Plastpolimer, St.-Petersburg, Russia) with an area of 20 cm^2^. One MFC device contained four series-connected graphite rods installed in the anode compartment and four rods in the cathode compartment. A schematic diagram of MFC operation and external connections is presented in Figure 1. A 25-mM PBS, pH 6.5, containing 10 mM sodium chloride, was used as anolyte and catholyte; 2,6-Dichlorophenolindophenol (DCPIP, 0.14 mM) in the anode chamber and potassium hexacyanoferrate (III) (4 mM) in the cathode chamber were used as redox mediators. As carbon sources for *G. oxydans* cells in the anode chamber, use was made of ethanol (within the concentration range from 0.05 up to 10 mM), glucose at concentrations of 1 or 3 mM, glycerol at a concentration of 1 mM or else municipal wastewater. The total area of the bioanode coated with biomass was 12 cm^2^ in one MFC device. The MFC operation was monitored at 25 ± 5 °C and constant stirring (500 rpm) for 21 days. A boost converter based on a bq 25,504 integrated circuit (Texas Instruments, Dallas, TX, USA) which performs direct current transformation, was used to increase MFC output voltage [36]. A low input voltage from the MFC was fed to the converter input to be amplified. Then the AC voltage produced in the secondary winding of the transformer was boosted once again and was rectified using an external charge-pump capacitor and internal rectifiers. The generated boosted electric energy was converted into a DC output of 3.2 V and was accumulated at a 6800-µF capacitor.

The chemical oxygen demand (COD) of wastewaters was assessed according to the Federative Environmental Normative Documents of the Russian Federation [37].

### 2.4. Electrochemical Characterization

The electrochemical characterization of the MFC was carried out by cyclic voltammetry and electrochemical impedance spectroscopy using a VersaSTAT 4 potentiostat galvanostat (Ametek Inc., Berwyn, PA, USA). The MFC anode was used as the working electrode; the cathode served as the reference as well as the counter electrode. Cyclic voltammograms were registered at a scan rate of 3 mV/s. Impedance measurements were carried out at applied potentials of 200 mV within the frequency range of 40 kHz to 0.02 Hz with an AC signal of a 10-mV amplitude. The Nyquist diagrams within the frequency range lower than 0.02 Hz were obtained by fitting the data using ZSimpWin (EChem Software, USA). This software was also used for picking the suitable equivalent circuit for every system. The measurements were conducted at a constant stirring of solutions.

The MFC power characteristics, internal resistances and Coulomb efficiency were calculated by the formulas from [38]. Power density (*P*) was determined according to the formula:

(1) $$P=\frac{I\times U}{S},$$

where *P* is the MFC power; *I*, the generated current; *S,* anode surface area; *U*, applied voltage. The current and voltage values were derived from the current–voltage characteristics. The MFC internal resistance was calculated as

(2) $$R_{in}=\frac{U^{2}}{P};$$

the Coulomb efficiency, as

(3) $$C_{E}=\frac{M_{S}\int_{0}^{t_{b}}I\;dt}{Fb_{es}v_{An}\Delta C}.$$

In Formula (3), *M*_S_ is the molecular weight of substrate, g/mol; Δ*C* is the substrate concentration change during the MFC operation, g/L; *F*, Faraday constant, 96,485 C/mol; *b*_es_, the number of electrons exchanged per mole of substrate (mol e^−^/mol); *v*_An_, the volume of the MFC anode compartment, L. To assess the MFC Coulomb efficiency in wastewater treatment, we used ΔCOD instead of ΔC, which is the difference between COD_in_ and COD_out_ (values in g/L).

### 2.5. Raman Spectroscopy

Graphite electrodes used for electrochemical studies served as substrates for Raman spectroscopy samples. The coating was sufficiently dense for D and G bands characteristic of the graphite substrates not to be present. The examination was carried out on a HEDA Raman spectrometer (NOST, South Korea), 532-nm Nd:YAG laser, grating 1200 grooves per mm (2.4 cm^−1^ per pixel) and matrix cooled to –50 °C, power, 0.6 mW (1% of 61.5 mW) on an area of ~1 μm^2^. The total spectrum was a sum of five 1 s measurements. The raw data were baseline corrected, and the fluorescence background was removed to yield the spectra shown in the subsequent figures.

## 3. Results

### 3.1. The Structure of the Composite

Earlier, we proposed a method for the immobilization of acetic acid bacteria on graphite screen-printed electrodes using a PEDOT:PSS/graphene/Nafion composite. These electrodes were used as part of a glucose biosensor [29]. In this work, the developed composite was used to create a microbial fuel cell based on *Gluconobacter oxydans* bacteria for wastewater treatment.

**Figure 2:**
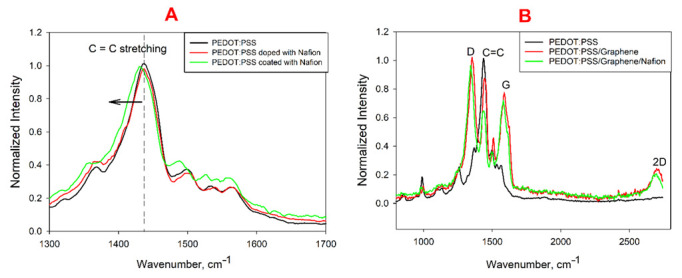
Raman spectra of PEDOT:PSS (**A**) or PEDOT:PSS/graphene (**B**) upon addition of Nafion.

PEDOT:PSS films coated and doped with Nafion on a graphite electrode surface were studied using Raman spectroscopy. Raman spectra (Figure 2A) show peaks characteristic of this compound, associated with deformation and asymmetric vibrations of the polymer chain structure [39]. The 1436 cm^−1^ broader peak, which is a convolution of two separate symmetrical Ca−Cß(−O) stretching modes dependent on a benzoid or quinoid PEDOT structure [40], was strongly pronounced. At the modification of PEDOT:PSS by Nafion, this peak shifted towards lower frequencies. It should be noted that this effect was weakly pronounced during the doping of the polymer with Nafion and during the coating the shift was 6 cm^−1^ (from 1436 cm^−1^ to 1430 cm^−1^). This indicates that the proportion of thiophene quinoid structures in PEDOT chains increased compared with benzoid structures at the introduction of Nafion [41]. The observed shift confirms once again that PEDOT chains bind to Nafion sheets. The quinoid-rich structure is a typical case of electron-rich or highly doped PEDOT chains, and the observed Raman shifts may arise from a change in their structure [42].

Figure 2B shows the spectra of PEDOT:PSS films modified by graphene and Nafion. The presence of graphene on the electrode leads to the emergence of characteristic pronounced peaks at ~1352 and ~1587 cm^−1^ (for D band and G band, respectively), as well as a 2D peak at a wavelength of about 2700 cm^−1^. Moreover, a pronounced C=C peak at 1436 cm^−1^, characteristic of PEDOT, shifts by 6 cm^−1^ towards high frequencies, which is indicative of an increase in the proportion of benzoid structures in the polymer. The Nafion coating leads to a reverse shift.

### 3.2. Electrochemical Characterization of the Biocomposite

**Figure 3:**
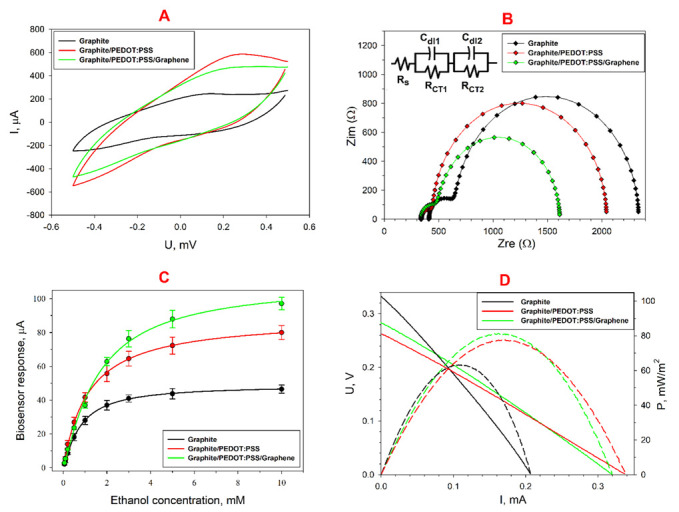
Electrochemical characteristics of the biocomposite modified with various types of PEDOT:PSS: (**A**) CVA of bioanodes at a scan rate of 3 mV s^−1^; (**B**) Nyquist plots for bioanodes; (**C**) calibration curves for ethanol for various types of bioanodes; (**D**) typical polarization curves obtained from cyclic voltammograms and power density curves.

Addition of electrochemically exfoliated graphene into the structure of PEDOT:PSS is likely to improve the polymer conductivity by forming a conductive network between graphene flakes [43]. The inter-stacking of graphene layers between PEDOT and PSS helps to delocalize charges in the PEDOT backbone and rapidly transfer electrons to DCPIP, thus reducing the charge-transfer resistance [44]. Improved conductivity can also be attributed to the lesser presence of insulator PSS particles in the conductive composition [45]. Figure 3A shows cyclic voltammograms for bioanodes with different coatings. As can be seen from the data obtained, the highest anode currents were observed for a composition with PEDOT:PSS. The CV of the PEDOT:PSS electrode is of a nearly rectangular form, and only one redox shoulder is present at 0.26 V indicating a low faradic activity and highly capacitive behaviour [46]. Addition of graphene to the conductive layer leads to a decrease in the capacitive component of the system, which should have a positive effect on the performance of this bioanode in the MFC. To assess the conductivity change of bioelectrodes based on various composites, we used electrochemical impedance spectroscopy. Figure 3B shows Nyquist plots and corresponding equivalent circuits for bioanodes with PEDOT:PSS and PEDOT:PSS/graphene. Addition of PEDOT:PSS and graphene was shown to lead to a decrease in the diameter of both semicircles in the diagrams, which corresponds to a decrease in the overall electron transfer resistance at the introduction of highly conductive components to the composite. The *R*_s_ value for the three electrodes was 355 ± 33 Ohms. The value of *R*_CT1_ at the addition of PEDOT:PSS decreased from 502 ± 21 to 224 ± 11 Ohms, and that of *R*_CT2_ at the addition of PEDOT:PSS with graphene decreased from 2042 ± 103 to 1021 ± 55 Ohms. Thus, it can be concluded that the addition of PEDOT:PSS/graphene has a positive effect on the overall conductivity of the bioanode in the MFC. We obtained calibration curves for substrate concentrations for the bioanodes formed (Figure 3C). Ethanol was taken as a substrate as it is utilized by *Gluconobacter* bacteria the most actively [27]. The sensitivity of the electrode to ethanol was maximal for the graphite/PEDOT:PSS composition to make 16.9 µA·mM^−1^·cm^−1^ and the detection range 0.05–10 mM. The Hill coefficient in all three cases was approximately the same and equal to unity, which indicates that the addition of PEDOT:PSS does not lead to a change in the cooperativity of substrate binding by bacterial enzyme systems. The values of the main parameters for all bioanodes are shown in Table 1. The polarization and power density curves recorded for the three MFC prototypes are shown in Figure 3D. It is worth noting that the maximum open circuit voltage (OCV) was observed in the MFC based on the control graphite bioanode; herewith, addition of conductive materials led to its decrease. The figure shows that the maximum power of 81 mW/m^2^ was obtained for the MFC based on a PEDOT:PSS/graphene-modified bioanode, which is comparable with the power of the PEDOT:PSS-based MFCs described in the literature [47,48].

### 3.3. Wastewater Treatment

**Figure 4:**
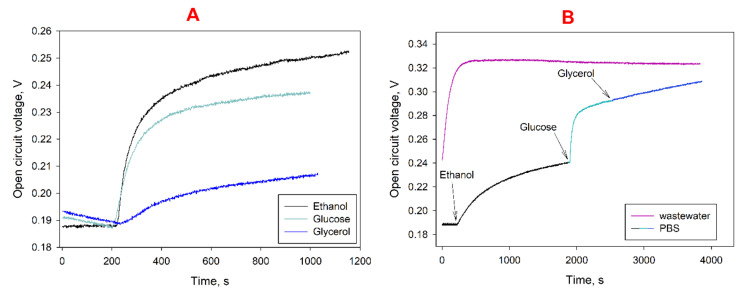
Change of MFC open circuit voltage at the introduction of three separate substrates into anolyte (**A**) or when using PBS with the sequential addition of substrates/municipal wastewater as anolyte (**B**). Concentrations of added substrates, 1 mM.

The strain *G. oxydans* has a wide substrate specificity [49] and is capable of oxidizing sugars, lower and triatomic alcohols and some organic acids [50]. We tested the possibility of generating energy by the developed MFC using three types of compounds as the sole carbon source. Glucose, ethanol and glycerol were picked, as all these compounds may be present in municipal wastewater. A change in the MFC open-circuit voltage at the addition of substrates into the anode compartment is shown in Figure 4A. Immediately upon addition of substrate into the MFC chamber, the OCV sharply increased; in the first seconds after addition, the rate of voltage change was 0.7 mV/s for ethanol, 0.4 mV/s for glucose and 0.06 mV/s for glycerol. After 15 min, the OCV level reached 90% of the generated maximum. The presence of several substrates in the MFC anolyte should lead to an overall increase in the generated MFC voltage. The change in OCV during the sequential addition of the investigated substrates to the MFC anode compartment is shown in Figure 4B. The voltage generated by the MFC increases from 0.25 V when using ethanol as a single substrate to 0.31 V when ethanol, glucose and glycerol are sequentially introduced into the cell at a concentration of 1 mM. The rate of OCV change in the first minutes of MFC operation was 0.5 mV/s, which is comparable to the rate at the addition of a separate substrate. The observed effect is due to the fact that several enzyme systems of microorganisms are simultaneously involved in this case. When the MFC bioanodes were immersed into a sample of wastewater taken at the Pushchino water treatment facilities, there was also a sharp OCV increase in the first minutes of operation. Figure 4B shows that the generated voltage when using wastewater without additional substrates turned out to be higher than for an MFC device based on PBS with three separate substrates added. Thus, it can be concluded that the wastewater sample under study contained easily oxidized organic compounds that can be utilized by *G. oxydans* immobilized on the MFC bioanode. This indicates that the developed MFC can be used for efficient wastewater treatment and generation of electricity in the process of the treatment.

**Figure 5:**
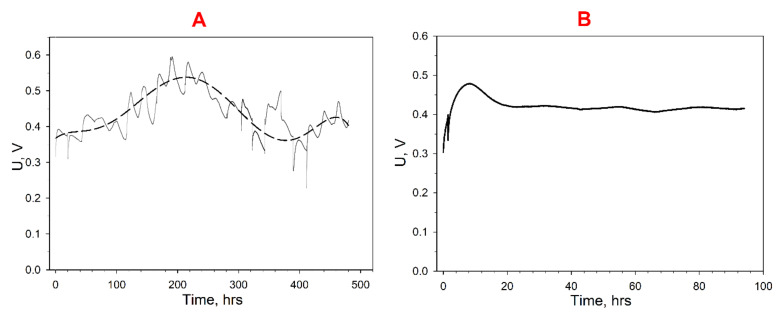
Long-term generation of electric energy by two series-connected MFCs based on PEDOT:PSS/graphene and during the oxidation of organic substrates contained in synthetic (**A**) and municipal (**B**) wastewater samples.

To assess the efficiency of the developed MFCs, the level of generated voltage was measured for several days. Both synthetic wastewater (with a glucose content of 3 mM) and real samples of municipal wastewater were used as an anolyte. When using synthetic wastewater, carbon sources were periodically depleted, so every four days 3 mM of glucose was added to the MFC anode compartments. Moreover, decolorization of mediator solutions in the anode and cathode compartments was observed due to the incomplete reversibility of the oxidation–reduction of these compounds (as seen by the asymmetry of the oxidation–reduction peaks on the CV (Figure 4A)). For this reason, redox mediators were additionally introduced into the MFC chambers every seven days (DCPIP and potassium hexacyanoferrate into the anode and cathode compartments, respectively). The obtained plots of MFC-generated voltage versus time are shown in Figure 5.

As is known from the literature data, most MFCs achieve maximum efficiency several days after they start to be operated [51]. The maximum levels of MFC-generated voltage based on synthetic wastewater (Figure 5A) were observed about a week after the start of work. After 10 days of MFC operation, the level of generated voltage was observed to decrease gradually. The maximum voltage during the entire MFC operation was 550 mV; the minimum, 340 mV. On day 8, after the MFC maximum efficiency was achieved, synthetic wastewater was replaced with municipal wastewater. The maximum voltage generated by the MFC operated on real wastewater was 480 mV (Figure 5B). Table 2 shows the Coulomb efficiency and COD removal rate for the developed MFC in the treatment of synthetic and municipal wastewater. The MFC operating time was taken to be 96 h (the interval between the introduction of fresh portions of anolyte). The Coulomb efficiency was shown to be higher during municipal wastewater treatment (39.8% vs. 16.5%), which is due to the large number of oxidizable substrates in the anolyte. A sufficiently high Coulomb efficiency can also be associated with the use of potassium hexacyanoferrate in the MFC cathode compartment [52]. Herewith, it is worth noting the relatively low COD removal rate during the treatment of real wastewater samples. These parameters are typical of pure culture-based MFCs [53,54], so in the future it would be advisable to use *G. oxydans* cells as part of a consortium in the anode department of an MFC for wastewater treatment. The use of fermentation wastewater containing sugars and alcohols as energy sources makes it possible to increase the COD removal rate since *G. oxydans* effectively oxidizes these substrates. Herewith, it should be noted that the wastewater used in this work had a pH = 7.9, and *G. oxydans* cells work most effectively at pH = 5.5–6.5 [50]. Thus, these microorganisms can also be used in treatment of wastewater with reduced pH. The data obtained were compared with the parameters of other pure culture MFCs presented in the literature (Table 2). As can be seen from the data of the table, the MFC operation time was from 1.5 up to 360 h (in our work, 96 h), which is indicative of the efficiency of bioreceptor immobilization on the electrode surface and implies that the used composite creates a favourable environment for cells and provides adequate pH conditions for their activity. Herewith, the maximum value of the voltage obtained in this work was 480–500 mV, and the power density, 65–82 mW/m^2^, which is comparable with similar parameters for the devices indicated in the table.

### 3.4. Accumulation of Electric Energy

As a rule, the scope of using MFCs as a power source is limited by their low power output. Moreover, the power supply of any MFC-based sensors is associated with the wireless transmission of data from the sensor over long distances, which requires even more energy [61]. The use of MFCs in practical applications requires either an increase in the area of the electrode or an increase in the number of MFCs connected in parallel. There is yet another method associated with the possibility of increasing the amount of energy using special converter devices.

The developed MFCs were connected in series and used to accumulate electricity using a DC/DC boost converter in synthetic and municipal wastewater treatment. With synthetic wastewater used in the first day of operation, two MFC models were able to charge a capacitor of 6800-µF capacity to 3.2 V in about 2 h. After 8 days of continuous operation, the MFC models charged the same capacitor to 3.2 V in just 7 min. The maximum efficiency of electricity generation was maintained for a week (on the 14th day, the capacitor charge time was still 7 min). Subsequently, the capacitor charge rate gradually decreased, and on the 20th day the charge time was about 55 min.

**Figure 6:**
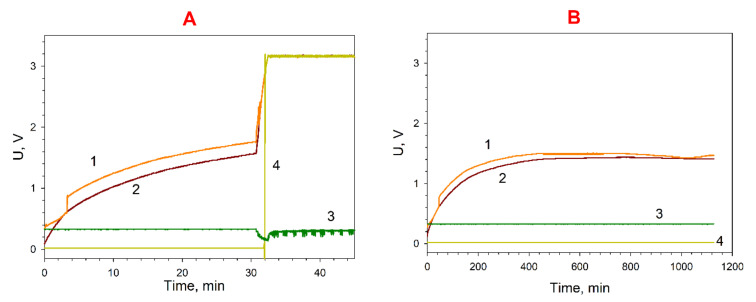
Diagrams of electric energy accumulation by a 6800-µF capacitor from two MFC models during wastewater treatment: 1, charge voltage; 2, voltage across the accumulating capacitance; 3, input voltage coming to the converter; 4, voltage signalling that the set charge level has been reached. (**A**), the first 24 h; (**B**), after 96 h.

Moreover, capacitors of 6800-µF capacity were charged using samples of municipal wastewater from the Pushchino water treatment facilities as an anolyte of two MFCs connected in series. Figure 6A shows a diagram of electric energy generation at an addition of electron transport mediators to the anolyte and catholyte. The average charge time of a 6800-µF capacitor when generating electricity from wastewater samples was 32 min. After 96 h of operation of the MFC on municipal wastewater, the level of harvested energy reached 1.5 V, but this was not enough to fully charge the capacitor, which indicates a decrease in the organic matter content in the anolyte (Figure 6B).

## 4. Conclusions

The paper presents an MFC prototype based on a pure culture of acetic acid bacteria *Gluconobacter oxydans*, which can be used for municipal wastewater treatment. The earlier developed composite based on a PEDOT:PSS conductive gel with graphene and Nafion was used to immobilize a biocatalyst on the surface of graphite anodes. The developed MFC generated a maximum voltage of about 550 mV with a current density of 2.1 mA/cm^2^ when using synthetic wastewater and a voltage of 480 mV with a current density of 1.8 mA/cm^2^ during the treatment of real samples of municipal wastewater. Herewith, the Coulomb efficiency of MFCs increased to 40%, and the COD removal rate was 32%. The MFC maximum power density of ~81 mW/m^2^ was achieved within days 8 to 15 of the operation. Thus, the composite we used provides charge transfer, as well as diffusion of substrates and reaction products, respectively, to and from microbial cells. The use of *G. oxydans* cells made it possible to provide for a sufficiently high COD removal rate in the case of using glucose, as well as for Coulomb efficiency, whose values are comparable with the analogs presented in the literature. Two parallel-connected MFCs were used to accumulate electric energy using a DC/DC boost converter and to charge a capacitor of 6800 µF capacity to 3.2 V in 7 min. Thus, the proposed system can provide additional power for a system of wireless sensors monitoring wastewater parameters during their treatment and be used as part of the Internet of Things technology. *G. oxydans* cells can also become an important part of MFC microbial consortia used for wastewater treatment of fermentation plants or wastewaters with low pH.

## Figures and Tables

**Table 1:** Characteristics of the bioanodes of MFCs, used as biosensors for ethanol detection.

	Electrode Modification	Control	PEDOT:PSS	PEDOT:PSS/Graphene
Parameter				
$I_{max}$, µA		48.8	88.6	109.9
Sensitivity, µA/mM		34.6	50.6	40.3
*K*_M_		0.77	1.14	1.58
*h*		1.18	1.01	1.16
Linear detection limit, mM		0.05–0.50	0.05–0.50	0.05–1.00
Minimal detection limit, mM		0.05	0.05	0.05
Detection range, mM		0.05–10	0.05–10	0.05–10

**Table 2:** Comparison of performances of various MFCs during the treatment of synthetic and real wastewater samples.

Anode Compartment	Carbon Source	Operation Time, h	Maximum Voltage, mV	Power Density, mW/m^2^	Coulomb Efficiency, %	COD Removal Rate, %	Reference
Carbon felt/*Rhizobium anhuiense*	Glucose	140	635	2.59	-	-	[55]
Graphite felt/*Bacillus subtilis*	Chicken manure	168	262	207.1	-	81	[56]
Carbon cloth/*Pseudomonas aeruginosa*	Dairy wastewater	360	1025	105	37.1	96	[57]
Carbon felt/*Shewanella. Baltica*	Artificial wastewater	1.5	190	12	-	57	[58]
Graphite plate/*Saccharomyces cerevisiae*	Dairy wastewater	264	850	-	-	92	[59]
Carbon fiber/*Escherichia coli*	Glucose	18	-	0.27 mW/cm^3^	21.3	-	[60]
Graphite rod/*Gluconobacter oxydans*/PEDOT:PSS/Graphene/Nafion	Glucose (3 mM)	96	550	82	16.5	85	This work
	Wastewater (279 mg O_2_/L)	96	480	65	39.8	32	
